# A scoping review on the use of virtual patients for enhancing empathy in medical students

**DOI:** 10.1080/10872981.2025.2607825

**Published:** 2025-12-25

**Authors:** Rie Yamada, Kaori Futakawa, Satoshi Kondo, Kuangzhe Xu, Satoshi Okazaki

**Affiliations:** a Department of Adult Nursing, Faculty of Medicine, Academic Assembly, University of Toyama, Toyama-shi, Toyama-ken, Japan; b Department of Maternal Nursing, Faculty of Medicine, Academic Assembly, University of Toyama, Toyama-shi, Toyama-ken, Japan; c Department of Medical Education, Graduate School of Medicine, University of Toyama, Toyama-shi, Toyama-ken, Japan; d Center for Medical Education and Career Development, Graduate School of Medicine, University of Toyama, Toyama-shi, Toyama-ken, Japan; e Cyberspace Security University of China, Wuhan Lingangang Economic and Technological Development Zone, Wuhan, People's Republic of China; f Department of Intelligent Robotics, Toyama Prefectural University, Toyama-ken, Toyama, Japan

**Keywords:** Students, medical, empathy, virtual reality, scoping review

## Abstract

Virtual patients have been increasingly utilized in medical education to develop empathy in a structured and scalable manner. Compared with traditional methods such as clinical practice and standardized patients, virtual patients can offer reproducible, resource-efficient learning experiences. This scoping review maps the research on virtual patient-based interventions designed to foster empathy in medical students. It seeks to identify existing research gaps, including conceptual definitions of empathy, scenario design, instructional strategies, assessment methods, and outcome measures. The Joanna Briggs Institute’s scoping review methodology was followed, and the PRISMA-ScR guidelines for reporting were used. A comprehensive search was conducted in PubMed, CINAHL, ERIC, Web of Science, Scopus, and the Cochrane Library. Two independent reviewers screened all titles, abstracts, and full texts; a third reviewer resolved any discrepancies. Findings were presented narratively and in tabular form to highlight key insights and research gaps. Eighteen studies involving 1,920 medical students were included. The most common study design was single-arm pre-post pilot studies (*n* = 4, 21.1%), followed by randomized controlled trials (*n* = 2, 10.5%) and mixed-methods designs (*n* = 2, 10.5%). Five research gaps were identified: 1) lack of explicit definitions of empathy, 2) limited diversity in clinical scenarios, 3) absence of repeated virtual patient interventions, 4) limitations in assessment methods, and 5) insufficient evidence on the sustained outcomes of empathy. These findings offer important insights into the current state of medical education, where standardized curricula for empathy training remain underdeveloped. Future efforts should address these challenges by integrating virtual patients into instructional designs that effectively foster empathy in medical students. This review provides a foundation for developing and implementing educational programs that meet the needs of students seeking to enhance their empathic abilities and contribute to improved patient outcomes and prevent clinician burnout.

## Introduction

Clinical empathy is the ability to understand a patient’s situation, perspective, and feelings and communicate that understanding to the patient [1]. It is a core clinical competence and a defining element of physicians’ professional identity. Empathy enhances patient satisfaction [2], promotes symptom disclosure [3], improves diagnostic accuracy [4], and contributes to better health outcomes [4,5], thereby strengthening the physician–patient relationship [6,7]. It also increases physicians’ job satisfaction and professional commitment [8] while reducing burnout [9]. In contrast, a lack of empathy is associated with patient dissatisfaction and complaints [10–12], higher rates of medical errors [10,12], preventable deaths [10], and greater physician burnout [9]. The importance of empathy extends to medical students as well. Empathy in medical students enhances professionalism [13] and reduces burnout [14], whereas a decline in empathy hinders altruistic attitudes and the development of professional identity [15,16]. These findings highlight that for medical students, whose empathy tends to decline during training [17,18], educational approaches that foster empathy are essential for developing a strong professional identity and becoming competent physicians.

Empathy is a learnable skill [19]. Various educational methods have been employed to cultivate empathy among medical students, including narrative medicine–based education [20], communication skills training [21], reflective writing [22], Balint groups [23], and training with simulated patients (SPs) [24,25]. However, these approaches face challenges such as financial constraints [25,26], limited consistency and reproducibility of educational content [26], and difficulties in standardising facilitators.

To address these limitations, increasing attention has been given to the use of virtual patients supported by virtual reality (VR) technology. Virtual patients are interactive computer simulations that replicate authentic clinical scenarios [27] and provide standardised disease representations [28]. VR technology enhances realism and immersion, allowing learners to engage more fully with clinical situations. Moreover, virtual patients can safely simulate scenarios involving infection risks or ethical concerns while offering advantages in cost-effectiveness and consistency. Accordingly, virtual patients are considered a promising educational tool for overcoming the limitations of traditional approaches.

The effects of empathy education using virtual patients have shown mixed results. While some studies have reported positive outcomes [29–32], others found limited or no effects [29,33,34]. These inconsistencies may stem from a lack of consensus on the definition of empathy among medical students [35], as well as variations in clinical scenarios, educational design, and assessment methods. Although several reviews have examined empathy in medical students [36–39], no review so far as focused specifically on the use of virtual patients. Previous systematic reviews on virtual patients in medical education have addressed clinical reasoning [40,41] and communication skills [42,43] but not empathy. Furthermore, although a review exploring empathy among healthcare students using virtual patients has been conducted [44], it included nursing, pharmacy, and dental students in addition to medical students. Given that empathy levels differ among health–care students, depending on their academic discipline [45], no comprehensive review has focused exclusively on the use of virtual patients to foster empathy in medical students. Moreover, the overall structure of educational design and assessment methods remains unclear.

Building on a previously published review protocol [46], this scoping review aims to systematically map existing studies on the use of virtual patients to foster empathy among medical students. Specifically, it examines the content of clinical scenarios, characteristics of educational design, and methods used to assess empathy, to identify the gaps in research. The findings are expected to provide an academic foundation for developing standardised curricula for empathy education and to offer practical insights for integrating virtual patients into medical education, where empathy training remains insufficiently established [47,48].

## Methods

Among the several types of reviews, a scoping review was selected to address the research objectives. A scoping review encompasses a broad range of literature regardless of the study design or quality and aims to map the overall landscape of existing evidence while identifying knowledge gaps that warrant further investigation [49]. In contrast, a systematic review focuses on identifying and synthesising international evidence related to a specific research question to evaluate the effectiveness of interventions [50]. As for a traditional literature review, while it summarises and integrates existing findings to outline research trends, it often lacks methodological rigour and systematic procedures [51].

This review aimed to systematically map existing studies on the use of virtual patients to foster empathy among medical students and to identify research gaps. Our intention was not to determine the effectiveness of virtual patient interventions. Given the need to comprehensively capture trends and gaps across the literature without imposing restrictive criteria, a methodologically structured scoping review was deemed the most appropriate approach.

The review was conducted in accordance with the methodological guidance of the Joanna Briggs Institute (JBI) for scoping reviews [52]. Following the Population, Concept, Context (PCC) framework recommended by the JBI [52], the review focused on undergraduate medical students (Population), empathy (Concept), and educational interventions using virtual patients in medical education (Context). To ensure transparency and methodological rigour, the reporting adhered to the Preferred Reporting Items for Systematic Reviews and Meta-Analyses extension for Scoping Reviews (PRISMA-ScR) checklist [53] (Additional file 1).

### Eligibility and exclusion criteria

To refine the focus of this review, explicit eligibility criteria were established in accordance with the predefined PCC framework [52]. Eligible studies were those that examined educational interventions utilising virtual patients with the aim of fostering empathy in undergraduate medical students. Empathy levels among undergraduate healthcare students vary across disciplines [45,54]. Therefore, including students from other undergraduate health-related disciplines could compromise the consistency of the results. Consequently, studies involving undergraduate or graduate students from other health-related disciplines—such as pharmacy, nursing, or dentistry—were excluded. However, studies were retained if they included participants from multiple health professions and either disaggregated data specific to medical students or had a sample composed of at least 80% medical students.

Only studies that treated empathy as a primary outcome or explicitly defined construct were included. Empathy is considered a skill, distinct from affective responses such as sympathy or compassion [55]. Therefore, studies focusing solely on these emotional constructs were excluded.

We included virtual patient interventions explicitly designed for educational use in medical curricula. Applications created primarily for commercial, recreational, or entertainment purposes, without an educational intent in medical training, were excluded.

No restrictions were applied to the publication year. Previous studies have reported a marked increase in the development and use of virtual patient technologies, including virtual reality, in medical education after 2010 [29]. However, studies employing VR–based virtual patients were also published before 2010 [56]. Therefore, we conducted a comprehensive search including all publication years.

This review included peer-reviewed research articles published in academic journals. Grey literature—defined as documents that are not commercially published and have not undergone formal peer review—was excluded, as screening such sources is time-consuming and yields a low likelihood of identifying relevant studies [57]. Searches for grey literature were conducted using Google, Google Scholar, and Semantic Scholar; however, most results retrieved via Google were commercial websites, with few academic or government-related reports. Searches using Google Scholar and Semantic Scholar mostly returned studies already indexed in PubMed, resulting in substantial duplication. Moreover, existing scoping reviews on VR in medical education [29] and other related fields [58,59] also excluded grey literature and limited their scope to peer-reviewed publications. Based on these findings, this review excluded grey literature and limited its inclusion to peer-reviewed journal articles only.

This scoping review aimed to explore the following research questions:

RQ1: Which research designs have been reported in studies exploring the use of virtual patients to foster empathy in undergraduate medical education?

RQ2: How did studies that utilised virtual patients for empathy training among medical students define empathy as a concept?

RQ3: What features characterise the clinical scenarios embedded in virtual patient simulations aimed at promoting empathy among medical students?

RQ4: What design features and technological formats have been used in virtual patient systems intended to support empathy development among medical students?

RQ5: In studies using virtual patients to promote empathy in medical students, what methods were utilised to assess empathy gains, and when were these assessments conducted?

RQ6: Which outcomes related to empathy have been documented in virtual patient-based research involving medical students?

### Search strategy

Before conducting the scoping review, we undertook a total of 20 hours of consultation involving a medical librarian and all co-authors to re-evaluate the validity of the search strategy and PCC framework described in our previously published protocol [46]. Consequently, the methodological validity was confirmed, and no modifications were deemed necessary regarding the PCC domains.

The literature search was conducted across six electronic databases: PubMed, CINAHL, ERIC, Web of Science, Scopus, and the Cochrane Library. The search strategy was constructed based on three core domains—Population (undergraduate medical students), Concept (empathy), and Context (virtual patients)—using a combination of controlled vocabulary (e.g., MeSH) and keywords. The strategy was optimised for each database’s indexing structure and implemented using Boolean operators (AND, OR) (Additional file 2).

### Selection criteria

All retrieved records were managed in Zotero 6 (Centre for History and New Media, George Mason University, US), where duplicates were removed before importing the dataset into Rayyan [60] for screening. No records written in languages other than English were identified.

In the first stage of screening, two independent reviewers (the first author, RY, and the second author, KF) assessed titles and abstracts based on the inclusion and exclusion criteria. The reasons for exclusion were documented, and any disagreements between the reviewers were discussed. If consensus could not be reached, the third author (SK) independently screened the records. Any additional discrepancies were resolved through discussion among all three reviewers [29]. During the second stage of screening, the same two reviewers independently reviewed the full texts of the selected studies. Each study was evaluated according to the predefined criteria, and the reasons for exclusion were recorded. As in the first stage, any disagreements were discussed based on the documentation, and if consensus could not be reached, the third author (SK) independently reviewed the full texts to resolve discrepancies.

Although both the first (RY) and second authors (KF) are faculty members at a nursing university, they are actively involved in undergraduate medical education and have conducted multiple studies on medical students’ learning and empathy training. Their involvement in the screening process was justified by their expertise in educational research and empathy training, ensuring accurate evaluation of the studies without disciplinary bias.

To assess the reliability of the screening process, Cohen’s *κ* coefficient was calculated using SPSS version 29.0 for Windows (IBM Corp., Armonk, NY, USA). The *κ* values were interpreted according to established benchmarks: ≤0.20 (no agreement), 0.21–0.39 (minimal), 0.40–0.59 (weak), 0.60–0.79 (moderate), 0.80–0.90 (strong), and >0.90 (almost perfect) [61].

The inter-rater agreement between the two reviewers was *κ* = 0.75 for title and abstract screening (moderate agreement) and *κ* = 0.88 for full-text screening (strong agreement).

### Data charting

As this scoping review aimed to descriptively organise and categorise the landscape of existing research [53], we did not assess the methodological quality or risk of bias of the included studies. Instead, we proceeded directly to data extraction for the purpose of information synthesis.

The data extraction form was based on the preliminary version developed in our previously published protocol [46], but was structurally modified to align with the specific focus of the current review. Through multiple rounds of discussion among the co-authors, five items were revised or removed due to redundancy or conceptual ambiguity (Additional file 3).

Two reviewers (RY and KF) independently extracted information from the selected literature. Extracted data included how empathy was defined in medical education, the structure of virtual patient scenarios, technological modalities employed, frameworks for instructional design, methods and timing of empathy assessment, and reported outcomes. When interpretation discrepancies arose, the third reviewer (SK) participated in discussions to facilitate consensus.

### Data synthesis and expert validation

To organise and interpret the extracted data, we first categorised the included studies according to their research objectives, methodological frameworks, and primary findings, which are summarised in Table 1. Two reviewers (RY and KF) independently examined this table and engaged in five iterative rounds of analysis to refine the structure. During this process, additional tables were developed to capture definitions of empathy (Table 2), descriptions of clinical scenarios (Table 3), and methods and timing of empathy assessment (Tables 4a and 4b). Divergences in interpretation were resolved through discussion within the research team, leading to consensus among all authors. The synthesised data were subsequently summarised in narrative form, aligned with the six research questions.

**Table 1.** Summary of the findings.

Author (Year)	Country	Purpose	Research design	Population and sample size	Findings regarding medical students’ empathy
Alieldin et al. (2024) [47]	United States	To examine the effectiveness of Immersive VR for empathy training in medical education.	Mixed methods study	19 first-year medical students, 9 men and 10 women. The mean age was 22 years (range 21-29).	Following the VR experience, medical students showed a statistically significant increase in their Jefferson Scale of Empathy scores. VR also enhanced students’ empathy towards older adults by fostering perspective taking.
Lin et al. (2024) [62]	Taiwan	To investigate whether immersive VR can enhance empathy and change attitudes toward depression in medical students.	Randomised Controlled Trial	59 fifth-year and sixth-year medical students,32 men and 27 women. The age range was 22 to over 25 years, with most participants aged 24–25.	VR experience enhanced medical students’ ability to adopt others’ perspectives (perspective taking) and their attitude of compassionate care (compassionate care).
Rehl et al. (2024) [61]	United States	To examine the effects of VR on empathy toward opioid use disorder among osteopathic medical students.	Single-arm pre-post pilot study	48 osteopathic medical students: 1 st year (*n* = 5), 2nd (*n* = 21), 3rd (*n* = 15), 4th (*n* = 7). 10 men and 38 women. The mean age was 24.5 ± 2.1 years.	Participants’ empathy scores increased significantly after the VR experience compared to before the experience.
Bard et al. (2023) [63]	United States	To evaluate the feasibility and effectiveness of VR in fostering empathy and understanding of dementia among pre-clinical medical students.	Single-arm pre-post pilot study	150 second-year pre-clinical medical students. The mean age and sex were not reported.	This VR experience significantly enhanced both cognitive and affective components of empathy among medical students by deepening their understanding of the perspectives of individuals with dementia and their families, and by fostering emotional resonance with their experiences.
Thng et al. (2022) [64]	Singapore	To evaluate how virtual and physical simulations influence medical students’ empathy and learning experience.	Pilot Study	20 first year medical students, 9 men and 11 women. The mean age was not reported.	The VR component, by enabling students to embody the child’s perspective, significantly enhanced both their measured empathy and their self-reported ability to understand paediatric patients’ experiences.
Zare-Bidaki et al. (2022) [65]	Iran	To assess the effects of experiencing one session of VR simulation of psychosis on empathy and knowledge compared to visiting patients in psychiatric wards.	Cluster randomised controlled trial	144 2nd and 3rd year medical students. VR group: 35 men and 37 women. The mean age was 19.90 ± 1.47 years. Contorol group: 30 men and 42 women. The mean age was 20.38 ± 1.04 years.	The VR group had significantly more empathy toward patients than students who visited patients under supervision.
Dupuy et al. (2021) [66]	France	To train students’ abilities to conduct a psychiatric interview.	Not reported	35 fourth-year medical students, 17 men and 18 women. The average was 22 years.	Medical students demonstrated effective use of empathic communication skills during their interactions with the virtual patient.
Elzie et al. (2021) [67]	United States	To evaluate the effectiveness of VR in end-of-life care education, including its impact on knowledge, confidence, and empathy.	Single-arm pre-post pilot study	145 first-year medical students, 74 men and 71 women. The mean age was not reported.	Medical students demonstrated an increased level of empathy toward the terminally ill patient and his family members following the VR experience, as evidenced by qualitative analysis of their written reflections.
Dupuy et al. (2020) [68]	France	To evaluate a VPs^2)^ for training medical students in psychiatric interviews, focusing on depression diagnosis and empathic communication.	Not reported	35 fourth-year medical students, 17 men and 18 women. The average was 22 years.	Following the VPs, students demonstrated high verbal empathy scores, indicating enhanced empathic communication performance. Empathic communication was significantly better than symptom recognition, suggesting prioritisation of empathy in student responses.
Elzie et al. (2020) [69]	United States	To examine the potential of immersive VR in fostering empathy among medical students.	Single-arm pre-post pilot study	154 first year medical students. The mean age and sex were not reported.	84.2% of students reported a better understanding of what patients with terminal cancer experience.
McLaughlin et al. (2020) [70]	United Kingdom	To explore medical students lived experiences of a VR simulation of hearing impairment.	Qualitative study (hermeneutic phenomenology)	10 medical students in the first and second years. The mean age and sex were not reported.	VR experience enhanced students’ empathy and motivated them to improve future communication with hearing-impaired patients.
Dupuy et al. (2019) [71]	France	To validate a depressive VP prototype and assess students’ diagnostic and empathic communication skills.	Not reported	34 fourth-year medical students, 17 men and 17 women. The mean age was 22 years.	Medical students managed to be empathetic and keep an emotional distance during interaction, as they should be with a real patient.
Guetterman et al. (2019) [31]	United States	To examine the effects of a virtual human-based simulation on empathic communication with patients and between professionals.	Mixed methods study	206 second-year medical students. The mean age and sex were not reported.	Students in the MPathic-VR intervention group demonstrated significantly higher empathic communication skills on the OSCE^4)^ compared to the control group. Students with higher performance in MPathic-VR demonstrated increased awareness of nonverbal empathic expressions, such as facial expressions, gestures, and nodding.
Dyer et al. (2018) [72]	United Kingdom	To enhance medical students’ empathy toward older adult patients using immersive VR^1)^	Not reported	178 first-year medical students. The mean age and sex were not reported.	VR enhanced students’ understanding of age-related health problems and increased their empathy for older adults with vision and hearing loss or Alzheimer’s disease.
Kron et al. (2017) [26]	United States	Comparing advanced communication skills and learning experiences between medical students using MPathic-VR and those using a multimedia module.	Mixed Methods study with Randomised Controlled Trial	421 second-year medical students. Intervention group: 106 men and 104 women. Mean age was 25.4 ± 2.6 years. Control group: 17 men and 94 women. Mean age was 25.5 ± 2.9 years.	The simulation enhanced students’ readiness to engage in emotionally sensitive and empathic clinical dialogues.
Foster et al. (2016) [32]	United States	To teach empathic communication to first-year medical students using a VP and to evaluate their verbal empathy in a subsequent SP.	Randomised Controlled Trial	70 first-year medical students, 37 men and 33 women. The mean age was 23.0 ± 1.7 years. Empathy-feedback VP group *n* = 35, Backstory VP group *n* = 18, Control VP group *n* = 17.	The empathy-feedback VP group was rated as demonstrating the highest empathy in subsequent SP interviews, both by trained assessors using Empathic Communication Coding System (ECCS) and by SPs using the communication checklist.
Kleinsmith et al. (2015) [25]	United States	To examine whether early-stage medical students can produce empathetic responses to VPs, and to compare them with responses to SPs^3)^	Not reported	73 third-year medical students, 42 men (Mage = 25.52 ± 2.62) and 31 women (Mage = 24.52 ± 1.52).	Medical students demonstrated significantly higher empathic responses toward VPs compared to standing in the patient’s shoes SPs. Longer responses to VPs were associated with higher third party-rated empathy scores among medical students.
Cordar et al. (2014) [73]	United States	To examine whether adding back stories to VPs interactions enhances medical students’ empathy and communication skills.	Not reported	35 first year medical students. The mean age and sex were not reported. VPs with backstory group *n* = 18, VPs without backstory group *n* = 17.	Medical students in the VPs with back story group were more likely to offer encouraging, supportive, and/or empathetic statements.
Deladisma et al. (2007) [56]	United States	To examine whether students demonstrated comparable levels of empathy during interactions with a VP and a SP.	Not reported	84 second year medical students. The mean age and sex were not reported. VP group *n* = 51, SP group *n* = 33.	Medical students demonstrated nonverbal communication behaviours and responded empathetically to a VP.

1) VR: Virtual Reality. 2) VPs: Virtual Patients. 3) SPs: Standard Patients. 4) OSCE: Objective Structured Clinical Examination.

**Table 2.** Sources and conceptual components of empathy definitions.

Author (Year)	Definition source	Summary of empathy definitions	Empathy components		
			Cognitive^b^	Affective^c^	Behavioural^d^
Rehl et al. (2024) [65]	Cited^a^	Viscerally experiencing from another’s point of view.	✓^e^	✓	
Bard et al. (2023) [67]	Cited	Empathy can include both a cognitive (understanding) and an affective (feeling) domain. Cognitive empathy has been described as the ability to understand someone’s situation without making it one’s own. Affective empathy refers to the emotional reaction one has in response to the experiences or emotional states of others. Empathy has been described as an emotional response that stems from another’s emotional state or condition and that is congruent with the other’s emotional state or situation.	✓	✓	
Zare-Bidaki et al. (2022) [70]	Cited	A non-judgmental attitude and understanding of patients’ viewpoints and emotions, described as a dynamic interpersonal process depending on the relationship, and a multidimensional construct containing cognitive and affective components.	✓	✓	
Elzie et al. (2021) [64]	Cited	Cognitive and emotional aspects: the cognitive aspect refers to the ability to comprehend another person’s inner experience and the capacity to understand the world from the other person’s viewpoint, whereas the emotional aspect refers to the ability to share someone else’s experiences and feelings—vicariously experiencing the emotional experience of others, and correctly identifying one’s own corresponding emotional state, perhaps via self-reflection and insight.	✓	✓	
McLaughlin et al. (2020) [66]	Cited	Not just an intellectual phenomenon is also an embodied and subjective experience, entering another world’	✓	✓	
Alieldin et al. (2024) [47]	Cited	An ability to understand the patient’s perspective and emotions and to effectively communicate that understanding to the patient. It comprises cognitive, affective, and behavioural dimensions: cognitive empathy refers to understanding others’ emotions intellectually, while emotional empathy refers to sharing and mirroring emotions.	✓	✓	✓
Guetterman et al. (2019) [31]	Cited	A set of constructs that relate to the ‘response of one individual to the experiences of another’. Constructs include cognitive, emotional, and behavioural outcomes.	✓	✓	✓
Foster et al. (2016) [32]	Cited	A complex phenomenon, conceptualised as having an affective component (the ability to share emotional experiences), a cognitive component (understanding the emotions of another person), and a behavioural component (the clinician’s verbal and nonverbal expression of empathy toward the patient).	✓	✓	✓
Dupuy et al. (2021) [69]	Cited	A deliberate intellectual effort to get ‘inside’ the other, for a better understanding.	✓		
Elzie et al. (2020) [73]	Cited	Step into the shoes of others and see the world from their perspective.	✓		
Dupuy et al. (2020) [71]	Cited	Verbal empathy, referring to the physician’s ability to ‘help the patient express his/her symptoms’; and nonverbal empathy, corresponding to the physician’s ability to stay neutral and to show empathic listening.			✓
Dupuy et al. (2019) [63]	Cited	Verbal empathy refers to physician’s ability to ‘help the patient express his symptoms’; and nonverbal empathy, corresponding to physician’s ability to keep his ‘observance stance’.			✓
Cordar et al. (2014) [68]	Cited	A predominantly cognitive (rather than emotional) attribute that involves an understanding (rather than feeling) of experience, concerns and perspectives of the patient combined with a capacity to communicate this understanding.	✓		✓
Kleinsmith et al. (2015) [25]	Original	Primarily cognitive, involving understanding the concerns of another person, as opposed to feeling their suffering.	✓		
Lin et al. (2024) [72]	Original	A cognitive attribute rather than an affective or emotional one.	✓		
Thng et al. (2022) [62]	Original	To put themselves in the shoes of the young child, to empathise and understand how a child feels while being treated.	✓	✓	
Dyer et al. (2018) [74]	Not Reported	No explicit definition provided.			
Kron et al. (2017) [26]	Not Reported	No explicit definition provided.			
Deladisma et al. (2007) [56]	Not Reported	No explicit definition provided.			

a ‘Cited’ indicates the definition was referenced from prior literature; ‘Original’ indicates the definition was generated by the study authors.

b Cognitive: intellectually understanding another person’s perspective or emotional state.

c Affective: emotionally resonating with or sharing another’s feelings.

d Behavioural: expressing empathy through verbal or nonverbal actions (e.g., supportive statements, gestures, or listening).

e A check mark (✓) indicates that the component was explicitly included in the study’s definition of empathy.

**Table 3.** Summary of scenario characteristics in virtual patients.

Primary diseases	Associated symptoms/comorbidities			Social context					
	Neuropsychiatric symptoms^a^	Neurovegetative symptoms^b^	Physical comorbidities^c^	Family conflict^d^	Social support^e^	Social disconnection^f^	Disclosure^g^	Bereavement^h^	Lack of mobility^i^
**Psychiatric disease**									
Major depressive disorder (*n* = 6)	32^j^, 65, 67, 68, 69, 73	67, 69, 73	–^k^	65	–	67	–	32, 65, 67	–
Alzheimer’s disease (*n* = 1)	70	–	–	70	70	70	–	–	–
Schizophrenia (*n* = 1)	63	–	–	–	–	63	–	–	–
Opioid use disorder (*n* = 1)	–	–	–	62	62	62	–	–	–
**Chronic disease**									
Cancer (*n* = 4)	71	–	–	26, 31, 71	66, 71	–	26, 31, 66, 71	–	71
Sensory impairments (*n* = 2)	–	–	–	–	–	–	–	–	–
Diabetes (*n* = 1)	47	–	47	–	47	47	–	47	47
Cranial nerve injury (*n* = 1)	–	–	25	–	–	–	–	–	–
**Not reported**									
Paediatric injection and dental procedures (*n* = 1)	–	–	–	–	–	–	–	–	–
Abdominal pain (*n* = 1)	–	–	–	–	–	–	–	–	

a Neuropsychiatric symptoms include hallucinations, delusions, depression, anxiety, and decreased motivation.

b Neurovegetative symptoms include fatigue, changes in appetite or sleep, psychomotor retardation, and decreased energy levels.

c Physical comorbidities include diabetes-related peripheral neuropathy and cardiovascular disease, as well as ptosis associated with cranial nerve injury.

d Family conflict includes differences in values among family members and between the patient and their family.

e Social support includes support from family members, hospital staff, and hospice care teams.

f Social disconnection includes social withdrawal, social isolation, self-isolation, and distrust of others.

g Disclosure includes both the diagnosis of cancer and prognostic information.

h Bereavement includes the death of a spouse and the death of a relative.

i Lack of mobility includes impaired ambulation due to diabetic neuropathy and wheelchair use in terminal cancer.

j Number indicates reference number.

k Not reported in the article.

**Table 4a.** Quantitative measures and timing.

Instrument used	Measurement timing	
**1-1. Validated questionnaire**		
The Jefferson Scale of Physician Empathy—Student Version (JSE-S) (*n* = 4)	Pre–Post (*n* = 3)	25, 47, 72
	Pre–VPs—Post–Lecture—Post–VPs^a^	63
Jefferson Scale of Empathy for Health Professional Students (JSE-HPS) (*n* = 2)	Pre–Post	62, 73
Medical Student Interviewing Performance Questionnaire (MSIPQ) (*n* = 1)	Post only	32
Interpersonal Reactivity Index (IRI) (*n* = 1)	Pre–Post	73
**1-2. Researcher-developed and company-provided questionnaires**		
A researcher-developed scale questionnaire: self-reported (*n* = 6)	Pre–Post	70, 71
	Post 1 yr only	72
	During VPs intervention	54, 68, 69
A researcher-developed scale questionnaire: SP-reported (*n* = 2)	Post only	32, 67
Company-provided standardised surveys (Embodied Labs) (*n* = 2)	Pre–Post	66, 74
**2. Performance-based assessments**		
Empathic Communication Coding System (ECCS) (*n* = 3)	Post only	25, 32, 67
Objective structured clinical exam (OSCE) (*n* = 2)	Post 1–14 days only	26, 31
System-generated scoring system (MPathic-VR) (*n* = 2)	During VPs intervention	26, 31
A researcher-developed performance-based scale (*n* = 1)	Post only	56
**3. Facial emotion recognition**		
Facial expression recognition (Affectiva) (*n* = 2)	During VPs intervention	58, 69

a Pre: before VP intervention, Post–Lecture: after lecture, Post–VPs: after VP interaction.

To strengthen the interpretive rigour and practical relevance of the findings, six experts in medical and nursing education were invited to review the results. Their feedback supported the utility of the synthesised evidence in highlighting how virtual patient-based learning may foster empathy in medical students and in identifying practical strategies for empathy assessment.

## Results

### Study inclusion

**Figure 1.**
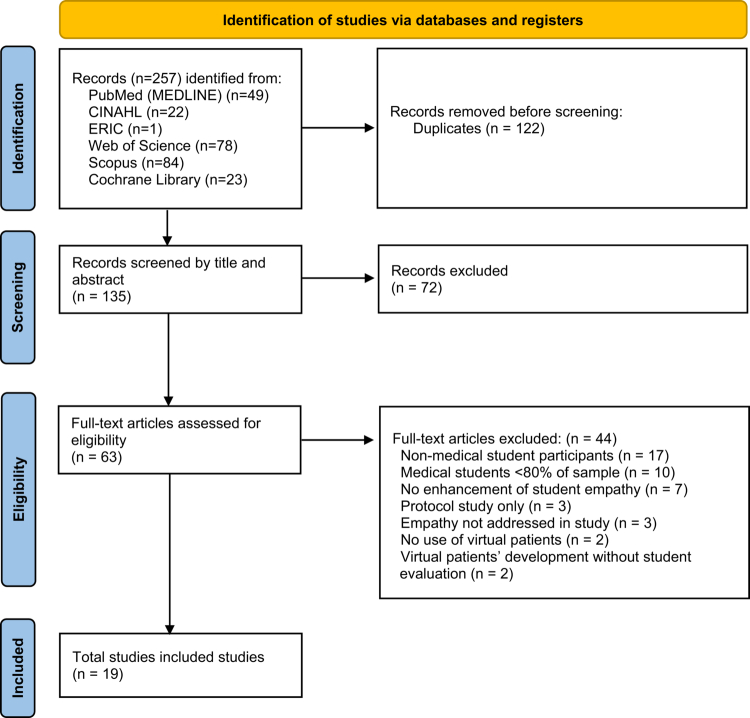
Stages of study identification and selection process.

The structured literature search was conducted from January 31 to February 12, 2025. A total of 257 records were retrieved from multiple literature databases: 49 from PubMed (MEDLINE), 22 from CINAHL, one from Education Resources Information Centre (ERIC), 78 from Web of Science, 84 from Scopus, and 23 from Cochrane Library. Figure 1 presents the search and screening process conducted based on the PRISMA 2020 flowchart [53]. After eliminating 122 duplicates, 135 records were screened based on titles and abstracts, leading to 72 exclusions. Among the 63 full-text records assessed for eligibility, 44 were excluded for the following reasons: non-medical student participants (*n* = 17), medical students comprising less than 80% of the sample (*n* = 10), no enhancement of student empathy (*n* = 7), protocol study only (*n* = 3), empathy not addressed in study (*n* = 3), no use of virtual patients (*n* = 2), and virtual patients’ development without student evaluation (*n* = 2) (Additional file 4). Consequently, 19 studies, all focusing on the use of virtual patients to enhance empathy among medical students, met the inclusion criteria and were included in the final review. A manual search of the reference lists of all 19 was also conducted, but no additional eligible studies were identified.

### Characteristics of included studies

The 19 studies (Table 1) were published between 2007 and 2024, with the following distribution: three in 2024, one in 2023, two in 2022, two in 2021, three in 2020, two in 2019, and one study each in 2018, 2017, 2016, 2015, 2014, and 2007. Most studies were conducted in the United States (*n* = 11), followed by France (*n* = 3) and the United Kingdom (*n* = 2), and there was one study each from Iran, Singapore, and Taiwan.

The total sample size across all studies was 1,920 with individual sample sizes ranging from 10 to 421. A mean age ranging from 19.9 to 25.5 years was reported in 10 of the 19 studies, indicating that participants were predominantly in their early twenties. All studies reported the academic year of the participating medical students. A total of 24 instances of year-level inclusion were reported across studies: first-year students (*n* = 9), second-year (*n* = 7), third-year (*n* = 3), fourth-year (*n* = 4), and fifth- and sixth-year students (*n* = 1 each).

### RQ1: which research designs have been reported in studies exploring the use of virtual patients to foster empathy in undergraduate medical education?

Of all 19 studies, four single-arm pre-post pilot studies were the most common (21.1%), followed by two randomised controlled trials (RCT) (10.5%) and one cluster RCT (5.3%). Two studies (10.5%) employed mixed-methods designs, and one (5.3%) used a mixed-methods approach incorporating an RCT. Other designs included one pilot study without pre-post comparison (5.3%) and one qualitative study using hermeneutic phenomenology (5.3%). Seven studies (36.8%) did not report on their research design.

### RQ2: how did studies that utilised virtual patients for empathy training among medical students define empathy as a concept?

Table 2 presents the sources and conceptual components of empathy definitions in the included studies. Among the 19 studies, 16 explicitly defined empathy, typically encompassing cognitive, affective, and behavioural components. Of these, 13 studies (68.4%) used cited definitions from previous literature [31,32,47,63–71,73], while only three (15.8%) used original definitions [25,62,72], and three (15.8%) did not report a definition [26,56,74] (Table 2).

Among the 13 studies that used cited definitions, five different patterns of empathy components were identified. Five studies defined empathy using both cognitive and affective components, making this the most common pattern [64–67,70]. Three studies adopted a triadic model, incorporating cognitive, affective, and behavioural elements [31,32,47]. Two studies defined empathy solely in cognitive terms [69,73], and another two focused exclusively on behavioural aspects [63,71]. One study defined empathy using a combination of cognitive and behavioural components [68].

Among the three studies with original definitions [25,62,72], two [25,72] conceptualised empathy primarily as a cognitive and perspective-taking process, explicitly distinguishing it from affective or emotional elements. In contrast, one study [62] defined empathy as a combination of cognitive understanding and emotional engagement, thus integrating both cognitive and affective components. Only two studies omitted cognitive components from their definitions, and no study defined empathy solely in affective terms.

### RQ3: what features characterise the clinical scenarios embedded in virtual patient simulations aimed at promoting empathy among medical students?

The virtual patient encompassed a wide range of clinical scenarios, with variations in age groups, primary diseases, associated symptoms or comorbidities, and social contexts. Fifteen studies (78.9%) reported the virtual patient’s age. Young adults (*n* = 6) [26,31,32,65,68,72] were most common, followed by older adults (*n* = 4) [47,64,66,74], middle-aged adults (*n* = 3) [63,67,71], one adult with unspecified age [70], and one paediatric case [62].

The primary diseases represented in the scenarios were categorised into three groups: psychiatric diseases, chronic diseases, and not reported (Table 3). Psychiatric diseases were reported in nine of the 19 studies (47.4%), with major depressive disorder being the most common (*n* = 6) [32,63,68,69,71,72]. Alzheimer’s disease [67], schizophrenia [70], and opioid use disorder [65] were each reported in one study (*n* = 1). Chronic diseases were identified in eight studies (42.1%). Cancer was the most frequently reported condition (*n* = 4), comprising leukaemia (*n* = 2) [26,31] and terminal cancer (*n* = 2) [64,73]. Sensory impairments, including age-related macular degeneration and hearing loss, were reported in two studies (*n* = 2) [66,74]. Diabetes [47] and cranial nerve injury [25] were each reported in one study (*n* = 1). Two studies (10.5%) [56,62] did not specify a diagnosis and focused on abdominal pain, paediatric injections, and dental procedures.

Primary diseases’ associated symptoms or comorbidities were categorised into three groups: neuropsychiatric symptoms, neurovegetative symptoms, and physical comorbidities. Neuropsychiatric symptoms were identified in 10 studies; these involved scenarios related to major depressive disorder (*n* = 6) [32,63,68,69,71,72], and one study each involved Alzheimer’s disease [67], schizophrenia [70], cancer [64], and diabetes [47]. Neurovegetative symptoms were reported in three studies, all of which focused on major depressive disorder [63,68,72]. Physical comorbidities were reported in two studies, involving diabetes [47] and cranial nerve injury [25].

The social contexts presented in the virtual patient scenarios were categorised into six domains: family conflict, social support, social disconnection, disclosure, bereavement, and lack of mobility. Family conflict was presented in six studies [26,31,64,65,67,69], of which three involved cancer [26,31,64]. Social support was presented in five studies [47,64,65,67,73], including two that presented cancer [64,73]. Social disconnection was presented in five studies [47,65,67,68,70], including four that presented psychiatric diseases such as major depressive disorder [65,67,68,70]. Disclosure was presented in four studies [26,31,64,73], all of which presented cancer. Bereavement was presented in four studies [32,47,68,69], including three that presented major depressive disorder [32,68,69]. Lack of mobility was presented in two studies [47,64], each of which presented diabetes [47] and cancer [64], respectively.

Among the 19 included studies, 17 scenarios presented psychiatric or other chronic diseases. Non-communicable diseases commonly encountered in humanitarian crises, severe infectious diseases, rare conditions, and acute illnesses requiring highly invasive treatments were not presented. Regarding associated symptoms and social contexts, physical symptoms were reported in only two studies each [25,47], while most scenarios focused on psychological and emotional aspects.

### RQ4: what design features and technological formats have been used in virtual patient systems intended to support empathy development among medical students?

Across the 19 included studies, virtual patient simulations were delivered using two primary technology modalities: immersive systems and desktop-based platforms. Ten studies used desktop-based or non-immersive platforms, including standard monitors or laptop computers [25,26,31,32,56,63,65,68,69,71]. The remaining nine studies employed immersive modalities, such as head-mounted displays or room-scale VR environments [47,62,64,66,67,70,72–74].

Regarding educational design, the duration of virtual patients’ interventions varied across studies. Six studies did not report the specific duration of the virtual patients’ session [31,56,63,66,73,74]. Further, one study reported a brief simulation lasting 4.5 minutes [70]. In total, seven studies implemented sessions ranging from 15 to 30 minutes [25,26,32,64,67,68,72], and three studies adopted simulations lasting between 30 and 60 minutes [62,69,71]. Additionally, two studies involved longer interventions of approximately 90 to 120 minutes [47,65].

In addition to differences in duration, some studies incorporated educational designs aimed at promoting empathy. These included virtual patients with background stories that provided psychosocial context [68] as well as simulations in which medical students were encouraged to offer empathic feedback during their interactions with the virtual patient [32]. All studies implemented the virtual patient simulations as a single-session intervention, and no study reported repeated exposure or longitudinal delivery.

### RQ5: in studies using virtual patients to promote empathy in medical students, what methods were utilised to assess empathy gains, and when were these assessments conducted?

Eleven studies used both quantitative and qualitative methods [26,31,47,62–64,67,69,71,73,74]. Seven assessed empathy exclusively using quantitative measures [25,32,56,65,68,70,72], while one study relied solely on qualitative methods [66].

In terms of quantitative assessments, three main categories were identified: questionnaire, performance-based assessments, and facial emotion recognition (Table 4a). The questionnaire included both validated questionnaires and researcher-developed and company-provided questionnaires. Among validated questionnaires, the most frequently used was the Jefferson Scale of Physician Empathy—Student Version (JSE-S) [75], which was employed in four studies [25,47,62,70]. This was followed by the Jefferson Scale of Empathy for Health Professional Students [76] in two studies [65,72], Medical Student Interviewing Performance Questionnaire [77] in one study [32], and Interpersonal Reactivity Index [78] in one study [72]. Researcher-developed questionnaires were used in eight studies [32,62–64,67–69,71], including six self-reported questionnaires [62–64,67,69,71] and two SPs-reported questionnaires evaluating medical students [32,68]. Additionally, two studies employed company-provided standardised surveys developed by Embodied Labs [73,74].

For performance-based assessments, the Empathic Communication Coding System [79], a validated observational coding system, was used in three studies [25,32,68]. In addition, two studies employed the Objective Structured Clinical Examination [26,31]. System-generated performance scores from MPathic-VR were used as a computer-based scoring method in two studies [26,31]. One study [56] employed a researcher-developed performance assessment tool.

Furthermore, two studies [63,71] utilised facial emotion analysis using Affectiva software to assess nonverbal empathic responses.

For qualitative assessments, semi-structured interviews were reported in five studies [47,63,66,69,71] and constituted the most frequently used method. This was followed by open-ended reflection responses in four studies [62,64,67,74] and reflective essays in two studies [26,31]. One study also employed a focus group interview [73] (Table 4b).

Regarding quantitative assessment timing, pre–post designs were the most commonly used, as reported in 10 studies [25,47,62,64,65,67,70,72,74]. Post-only assessments were conducted in seven studies [25,26,31,32,56,62,68], including three immediately after the intervention [25,32,68], three within several days to two weeks [26,31,56], and one with a three-point design including a one-year follow-up [62]. Additionally, five studies assessed empathy during the virtual patient intervention [26,31,63,69,71].

Regarding the timing of qualitative assessments, post-only evaluations were conducted in 11 studies [26,31,62–64,66,67,69,71–74]. Of these, 10 assessed empathy immediately after the virtual patient intervention, while one [62] conducted the assessment at a one-year follow-up. Additionally, one study [47] evaluated empathy at two time points: immediately post-intervention and again six months later.

Empathy in studies using virtual patients was primarily assessed through questionnaires and semi-structured interviews. In contrast, only two studies employed objective indicators, such as facial emotion recognition. Furthermore, only two studies longitudinally evaluated the sustained effects of virtual patient interventions on empathy.

### RQ6: which outcomes related to empathy have been documented in virtual patient-based research involving medical students?

Improvements in medical students’ empathy through the use of virtual patients were reported in all 19 included studies (Table 1). Regarding the timing of these improvements, 17 studies [25,26,31,32,56,63–74] reported enhanced empathy immediately following the virtual patient intervention, while two indicated that the effects were sustained at six months [47] and one year [62], respectively. The reported outcomes included improvements in empathy scores, enhancements in cognitive empathy, and increased awareness of the clinical significance of empathy.

Improvements in empathy scores were reported in 14 studies using either validated or researcher-developed instruments [25,32,47,62–65,67–72,74]. Among these, one study [62] found that JSE-S scores at one-year follow-up were significantly higher than baseline scores.

Five studies [47,64,66,67,73] demonstrated that virtual patient interventions contributed to the enhancement of cognitive empathy in medical students. Three of these [66,67,73] reported that immersive and realistic virtual patient programmes were effective in promoting perspective-taking. Two studies [47,64] showed that experiencing the emotions of virtual patients from a first-person perspective facilitated emotional understanding. One study [47] demonstrated that students retained empathy-related skills and recalled their virtual patient experience even six months after the intervention.

Four studies [26,31,62,66] reported an increased awareness of the clinical significance of empathy. The findings indicated that students recognised the role of empathy in improving the quality of clinical training [26,62,66] and became aware of the importance of nonverbal empathic communication, such as gestures and nodding [31].

Two studies employed facial expression recognition technology to capture nonverbal empathic responses; however, no clear association with empathy scores was reported [63,71]. Although all studies reported improvements in empathy, only two examined whether these effects were sustained over time, and none investigated whether enhanced empathy was applied in clinical practice after graduation. Moreover, studies that investigated associations between empathy and objective indicators did not identify any clear relationships.

## Discussion

This scoping review is the first to comprehensively map existing evidence on virtual patient interventions aimed at enhancing empathy in medical students. RQ1 examined the study designs of empathy education involving virtual patients. Although diverse designs were identified, no clear research gaps emerged. In relation to the remaining RQs, five major research gaps were identified across the 18 included studies: 1) lack of explicit definitions of empathy, 2) limited diversity in the clinical scenarios used in virtual patient interventions, 3) limited implementation of repeated virtual patient-based interventions, 4) the methodological limitations associated with the use of self-report questionnaires, and 5) insufficient evidence on the long-term application and sustainability of enhanced empathy.

### Lack of explicit definitions of empathy

Of the 18 studies included in this review, none defined empathy solely in terms of affective components, and only two definitions omitted cognitive elements [63,71]. The interpretation and application of terminology change over time [80]. Before 2000, clinical definitions of empathy were more strongly linked to emotion than cognition [81]. However, an over-emphasis on affective empathy can lead to loss of objectivity through over-identification [82] and heighten stress and burnout associated with emotional labour [4]. In the early 2000s, Hojat reconceptualized empathy as a primarily cognitive ability to understand and convey patients’ experiences, concerns, and perspectives [75], and attention to the cognitive dimension has since intensified [82]. The predominance of cognitively oriented definitions in the present review thus reflects this conceptual shift.

Only three studies employed investigator-generated definitions [25,62,72]. As empathy is multifaceted [75] and culturally contingent, achieving consensus on its definition has been challenging [83], and a unified definition is still lacking in medical education [35]. Nevertheless, a clear and explicit definition is essential for designing and evaluating effective interventions to enhance medical students’ empathy [83,84]. Educators should therefore first state the definition to be adopted; specify which cognitive, affective, or behavioural elements must be emphasised; and align curricular design and assessment metrics accordingly.

### Limited diversity in clinical scenarios

The primary diseases addressed in the scenarios were psychiatric diseases and chronic conditions; severe infections, rare diseases, and acute conditions requiring highly invasive interventions were not included. Associated symptoms and social context primarily focused on psychological distress, while acute physical symptoms and emergency situations were not represented.

For medical students with limited clinical experience, psychological burdens such as anxiety and reduced motivation associated with psychiatric and chronic conditions may be easier to understand, and such scenarios may serve as useful materials for fostering empathy. In fact, medical students are known to express empathy more readily toward patients experiencing anxiety, fear, or sadness [84].

In contrast, in acute or emergency situations, medical students tend to prioritise clinical reasoning and task prioritisation based on medical knowledge over empathic engagement 39,85. In emergency medical care, empathy has been shown to contribute to the prevention of adverse events and complications among vulnerable patients [86]; higher levels of provider empathy are associated with greater success rates in highly invasive treatments [87]. Conversely, lower levels of empathy are linked to higher rates of burnout among emergency care providers [88]. These findings suggest that empathy plays a crucial role in dealing with not only mental illness and psychosocially distressing contexts but also life-threatening acute conditions and situations requiring invasive treatments, with implications for both clinical outcomes and the mental health of healthcare professionals. Additionally, these results underscore the importance of enabling medical students to develop empathy in such contexts.

Virtual patients offer advantages in scalability, cost-effectiveness, reproducibility, and consistency [46]; they also provide a safe and flexible educational environment for learning about rare, severe, and high-risk clinical situations that are difficult to encounter during standard clinical training. Therefore, it is essential to develop virtual patient scenarios that encompass not only cases where students find it easier to express empathy but also a broader range of acute illnesses, emergencies, invasive procedures, and infectious diseases, thereby enhancing empathy across diverse clinical settings.

### Restricted implementation of repeated interventions

All studies included in this review implemented virtual patient simulations as a single-session intervention; no study reported repetitive or longitudinal implementation. Empathy in medical students tends to decline during clinical training, indicating the need for not one-off interventions, but rather, repeated and sustained educational strategies [89]. In other VR-based educational domains, such as language acquisition [90] and psychomotor training [91], repeated and temporally distributed practice has been shown to enhance memory retention and skill acquisition more effectively than single-session formats. These findings suggest that repeated and sustained implementation of virtual patient-based interventions may more effectively enhance empathy in medical students and potentially support the maintenance of empathic engagement in future clinical practice. Further studies are needed to compare single versus repeated interventions and determine the optimal frequency and duration of virtual patient-based programmes for fostering empathy in medical education.

### Limitations of self-report questionnaires for assessing empathy

In assessing empathy, self-administered questionnaires were predominantly used, with only two studies employing physiological measures. Similarly, only two studies assessed empathy longitudinally.

Even validated self-report questionnaires are susceptible to respondents’ subjective biases [92] and have limitations in fully capturing the multidimensional construct of empathy [93]. Furthermore, self-administered questionnaires may lead to respondent fatigue and reduced concentration as the survey progresses, potentially compromising the completeness and accuracy of responses [94]. To address these issues, increasing attention has been directed toward the use of physiological indices as objective measures of empathy. For example, heart rate variability [93], cross-entropy between micromovement signals [95], and eye movement [96] have been investigated as potentially useful, noninvasive, and objective indicators of empathic responses. These methods are considered suitable for educational settings as they allow for real-time recording of empathic responses while minimising the burden on learners. In particular, the introduction of noninvasive measures may be beneficial in educational programmes that aim to monitor changes in empathy on a regular and repeated basis. However, empathy is a complex construct comprising cognitive understanding, emotional resonance, and behavioural expression, and current physiological measures present challenges in terms of identifying which components they capture, as well as issues of feasibility and practicality in educational contexts.

Future research should focus on developing and validating physiological measures that can appropriately and practically assess the multidimensional nature of empathy. This establishes a foundation for longitudinal and multifaceted evaluation of the effects of virtual patient-based empathy education.

### Insufficient evidence on the long-term application and sustainability of empathy

Although all studies reported improvements in empathy, only two examined the sustainability of these effects over time [47,62], and none investigated whether enhanced empathy was applied in clinical practice after graduation. In virtual patient-based learning, educational content is encoded in association with specific environmental contexts. This improves memory retrieval when learners mentally reconstruct those contexts during recall [90]. Furthermore, VR provides learners with a heightened sense of presence, which has been shown to facilitate the encoding, retention, and retrieval of learning experiences [97]. The immersion and context reinstatement enabled by VR may contribute to the long-term retention of empathic attitudes and skills, as evidenced by sustained empathy scores at six months and one year post-intervention [47,62].

Empathy plays a vital role in not only preventing medical student burnout [14] and fostering professionalism [13] but also building trust with patients [6,7] and improving clinical outcomes [4,5]. Therefore, the enhancement of empathy through virtual patients should not be limited to immediate post-intervention gains but should ideally translate into sustained empathic behaviour in clinical practice after graduation. However, empathy education in medical curricula remains insufficient. In addition, factors such as academic overload, performance-based stress, and negative role modelling have been identified as barriers to empathy development among medical students [38]. Consequently, even if empathy is enhanced through virtual patient interventions, there remains a risk of decline over time owing to these educational and systemic constraints. Future research should longitudinally and comprehensively examine how empathy enhanced through virtual patients is maintained and operationalized in real-world clinical settings during and after undergraduate medical education. Virtual patients offer advantages such as scalability, consistency, and reproducibility and are considered a promising tool for empathy education. However, this review identified only 18 relevant studies, suggesting that the field is still in an early stage of development. The five research gaps must be accounted for in an integrated manner, according to the objectives of empathy education and the characteristics of the target students. If they are designed independently without interconnection, there is a risk of misalignment with educational goals and inconsistency in evaluation results.

In particular, empathy among medical students has been reported to decline as they progress through their studies [17,18]. Therefore, it is necessary to clearly define which components of empathy (cognitive, affective, behavioural) should be fostered and to select appropriate scenario designs, intervention frequencies, and assessment methods accordingly. For example, in junior students with relatively preserved empathy [17,18] and limited clinical experience, a single intervention based on a standard scenario involving common diseases and comorbidities may be effective. In contrast, senior students who have completed clinical clerkships are more likely to focus on clinical reasoning and procedural decision-making 39,85, leading to a decline in empathy [17,18]. Therefore, it may be more effective to implement multiple interventions using scenarios that involve complex clinical situations, such as high-acuity or rare diseases.

### Implications for medical education

The findings indicate two essential educational engagements required for faculty members as virtual patient–based empathy education becomes more widespread in undergraduate medical curricula.

First, educators must to carefully assess students’ readiness. Most existing virtual patient scenarios have dealt with conditions such as major depressive disorder [32,63,68,69,71,72] or cancer [26,31,64,73], which are relatively easy for novice students to understand in terms of disease mechanisms and patients’ emotional distress. This suggests that improvement in empathy requires students to be able to understand or imagine the disease depicted in the clinical scenario and comprehend its physiological context. Although it is theoretically possible to feel compassion without fully understanding the disease, such a response is closer to compassion than to clinical empathy. Therefore, as more diverse scenarios (e.g., rare or acute diseases requiring invasive interventions) develop, educators must be capable of assessing whether medical students possess the necessary readiness to understand and conceptualise the presented diseases, including their physiological and psychological aspects.

Second, feedback from educators after virtual patient use is indispensable. None of the 18 studies reviewed reported educator-led feedback following virtual patient sessions. While computer-generated debriefing can enhance short-term skill acquisition, sustained learning retention requires facilitator feedback from experienced instructors [98]. As empathy contributes to the development of professional identity and is fundamental to future clinical practice, it should ideally be maintained and reinforced over time rather than fading after initial improvement. Even if virtual patients are equipped with automated feedback functions in the future, the sustained enhancement of empathy still depends on reflective feedback provided by educators. Thus, faculty members must cultivate the ability to verbalise and convey their own empathic attitudes to students.

Third, novice medical educators should actively engage in medical education using virtual patients. According to [99], virtual patient–based education provides opportunities to carefully reflect on learning objectives and teaching methods, thereby enhancing educators’ educational competencies. By utilising virtual patients, novice educators can practically learn the processes of instructional design and assessment in empathy education. Therefore, virtual patient–based education can be considered one of the effective and practical teaching methods to enhance teaching competence among novice medical educators.

In summary, as virtual patients become increasingly integrated into medical education, educators’ engagement is considered essential in three areas: assessing learners’ readiness for clinically complex cases, providing reflective feedback that fosters empathy across cognitive, affective, and behavioural dimensions, and encouraging novice medical educators to actively utilise virtual patients.

### Limitations

This review has some limitations. First, the range of databases searched was limited, and some relevant studies may have been omitted. Future studies should consider using a broader range of databases and including grey literature to achieve a more comprehensive identification of relevant studies. Second, although this scoping review followed a rigorous methodology based on the JBI guidelines [52], the nature of scoping reviews precludes any quantitative evaluation of intervention effectiveness, causal inference, or effect size estimation. Therefore, the accumulation of RCTs and other intervention studies on this topic is necessary for enabling future systematic reviews that can quantitatively assess the effectiveness of virtual patient-based empathy education. Furthermore, study quality was not appraised, and no restrictions were placed on study design, which may affect the reliability and generalisability of the findings. Future reviews should specify study designs and conduct quality appraisals to improve the reliability of the findings.

## Conclusion

This scoping review is the first to comprehensively map existing evidence on virtual patient interventions aimed at enhancing empathy in medical students. Five key research gaps were identified across eighteen included studies: 1) lack of explicit definitions of empathy, 2) limited diversity in clinical scenarios used in virtual patient interventions, 3) limited implementation of repeated virtual patient-based interventions, 4) methodological limitations associated with the use of self-report questionnaires, and 5) insufficient evidence on the long-term application and sustainability of enhanced empathy. The development of virtual patients designed to address these research gaps can contribute to establishing standardised empathy education programmes and foster the development of empathic attitudes in future clinicians. Furthermore, medical educators and instructors are recommended to evaluate students’ readiness for complex clinical scenarios and enhance their own capacity to provide reflective feedback, as these practices are likely to maximise the educational impact of virtual patient–based interventions aimed at fostering empathy.

## Supplementary information

Supplementary information is available in an online document on OSF https://osf.io/bzndc/.

**Table 4b.** Qualitative assessment approaches and timing.

Data source/Method	Assessment timing	
Semi-structured interview (*n* = 5)	Post only	64, 65, 68, 69
	Post-6 month follow-up	47
Open-ended reflection responses (*n* = 4)	Post only	70, 71, 74
	Post-1yr only	72
Reflective essay (*n* = 2)	Post only	26, 31
Focus group interview (*n* = 1)	Post only	66

## Supplementary Material

Additional file 4.docx Additional file 4.docx

Additional file 2.docx Additional file 2.docx

Additional file 3.docx Additional file 3.docx

Additional file 1.pdf Additional file 1.pdf

## Data Availability

Not applicable.
