# Supplementary material for: A scoping review on the use of virtual patients for enhancing empathy in medical students
Source: Med Educ Online. 2025 Dec 25;31(1):2607825. doi: 10.1080/10872981.2025.2607825 (PMC12777885; doi:10.1080/10872981.2025.2607825)
Supplement: Additional file 4.docx [file ZMEO_A_2607825_SM7675.docx]

Additional file 4: Excluded studies in full-text review (n=44)

| # | Author (Year) | Title | Exclusion reasons |
| --- | --- | --- | --- |
| 1 | Jones et al. (2021) | Use of virtual and augmented reality-based interventions in health education to improve dementia knowledge and attitudes: an integrative review | Medical students <80% of sample. |
| 2 | Anderson et al. (2024) | Virtual reality education on myalgic encephalomyelitis for medical students and healthcare professionals: a pilot study | Medical students <80% of sample. |
| 3 | Quail et al. (2016) | Student self-reported communication skills, knowledge and confidence across standardised patient, virtual and traditional clinical learning environments. | Non-medical student participants. |
| 4 | Lem et al. (2022) | Effect of a virtual reality contact-based educational intervention on the public stigma of depression: randomized controlled pilot study | Medical students <80% of sample. |
| 5 | Pira et al. (2025) | Could empathy be taught? The role of advanced technologies to foster empathy in medical students and healthcare professionals: a systematic review | Medical students <80% of sample. |
| 6 | Torrence et al. (2023) | Preparing future health care workers for interactions with people with dementia: a mixed methods study | Non-medical student participants. |
| 7 | Ko et al.  (2018) | Involving volunteer patients VPs in medical education not only trains the medical students but also promotes the VPs’ health | No use of virtual patients. |
| 8 | Dyer et al. (2018) | Using virtual reality in medical education to teach empathy | Medical students <80% of sample. |
| 9 | Kane et al. (2024) | Managing emotional dialogue for a virtual cancer patient: a schema-guided approach | Empathy not addressed in study. |
| 10 | Jacobs et al. (2022) | Learning from 360-degree film in healthcare simulation: a mixed methods pilot | Medical students <80% of sample. |
| 11 | Kizhevska et al. (2022) | Using virtual reality to elicit empathy: a narrative review | Non-medical student participants |
| 12 | Erzsébet et al. (2024) | Artificial intelligence in health education: blessing or curse? | Non-medical student participants. |
| 13 | Takata et al. (2024) | Development of a virtual patient model for Kampo medical interview: new approach for enhancing empathy and understanding of Kampo medicine pathological concepts | VPs development without student evaluation. |
| 14 | Shah et al. (2010) | A pilot study to evaluate the use of an interactive virtual patient with depression to teach history-taking skills in a psychiatry clerkship | Empathy not addressed in study. |
| 15 | Abrams et al. (2024) | Virtual reality-based simulated hallucinations to enhance empathy toward individuals with schizophrenia | Non-medical student participants. |
| 16 | Dhar et al. (2023) | A scoping review to assess the effects of virtual reality in medical education and clinical care | Medical students <80% of sample. |
| 17 | Gilbert et al. (2024) | Using virtual patients to support empathy training in health care education: an exploratory study | Non-medical student participants. |
| 18 | Alexander et al. (2021) | Design of visual deficit simulation for integration into a geriatric physical diagnosis course | Medical students <80% of sample. |
| 19 | Wu et al. (2019) | Enhancing medical students’ communicative skills in a 3D virtual world | Medical students <80% of sample. |
| 20 | Meijer et al. (2020) | Exploring visuo-tactile embodiment in a social virtual reality setting with a physical wheelchair for training empathy towards social disability barriers | Protocol study only. |
| 21 | Li et al. (2021) | Parkinson’s disease simulation in virtual reality for empathy training in medical education | Medical students <80% of sample. |
| 22 | Corriette et al. (2023) | Using VR to elicit empathy in current and future psychiatrists for their patients of color | Non-medical student participants. |
| 23 | Henry et al. (2021) | Enhancing medical professionals’ and students’ empathy for visually impaired patients using virtual reality | Non-medical student participants. |
| 24 | Carnell et al. (2022) | Informing and evaluating educational applications with the Kirkpatrick model in virtual environments: using a virtual human scenario to measure communication skills behavior change | VPs development without student evaluation. |
| 25 | Tsekhmister et al. (2023) | Virtual reality in EU healthcare: empowering patients and enhancing rehabilitation | Non-medical student participants. |
| 26 | Marques et al. (2022) | Impact of a virtual reality-based simulation on empathy and attitudes toward schizophrenia | Non-medical student participants. |
| 27 | Gugliucci et al. (2021) | End of life virtual reality training: medical student increased empathic ability | Non-medical student participants. |
| 28 | Sapkaroski et al. (2022) | Immersive virtual reality simulated learning environment versus role-play for empathic clinical communication training | Non-medical student participants. |
| 29 | Tong et al. (2020) | Designing a virtual reality game for promoting empathy toward patients with chronic pain: feasibility and usability study | Non-medical student participants. |
| 30 | Virginia Commonwealth University. (2019) | Embodied empathy; virtual reality and experiencing geriatrics | Protocol study only. |
| 31 | Author unknown. (2024) | Preliminary research of empathy education in medical students through virtual scenario experience | Protocol study only. |
| 32 | Toscano. (2013) | Toward a full integration of teaching/learning professionalism and clinical competence in medical students | Empathy not addressed in study. |
| 33 | Mahling et al. (2023) | Virtual Reality for Emergency Medicine Training in Medical School:  Prospective, Large-Cohort Implementation Study | No enhancement of student empathy. |
| 34 | Washington et al. (2019) | The effects of a VR Intervention on career interest, empathy, communication skills, and learning with second-year medical students | No enhancement of student empathy. |
| 35 | Duke et al. (2014) | Preserving third year medical students’ empathy and enhancing self-reflection using small group ‘‘virtual hangout’’ technology | No enhancement of student empathy. |
| 36 | Zielke et al. (2017) | Developing Virtual Patients with VR/AR for a natural user interface in medical teaching | Non-medical student participants. |
| 37 | Loue (2022) | Teaching and practicing humanism and empathy through embodied engagement | No enhancement of student empathy. |
| 38 | Newcomb et al. (2021) | Building Rapport and Earning the Surgical Patient's Trust in the Era of Social Distancing: Teaching Patient-Centered Communication During Video Conference Encounters to Medical Students | No enhancement of student empathy. |
| 39 | Kohn et al. (2025) | Virtual reality communication training in pain medicine: effects on medical students’ racial bias, empathy, and interview performance with virtual patients | No enhancement of student empathy. |
| 40 | Stewart et al. (2023) | Training for psychiatric assessments using virtual simulation. | No enhancement of student empathy. |
| 41 | Lee et al. (2020) | Effective virtual patient simulators for medical communication training: A systematic review | Non-medical student participants. |
| 42 | Abdool et al. (2017) | Simulation in undergraduate psychiatry: exploring the depth of learner engagement | No use of virtual patients. |
| 43 | Chaby et al. (2022) | Embodied virtual patients as a simulation-based framework for training clinician-patient communication skills: an overview of their use in psychiatric and geriatric care | Non-medical student participants. |
| 44 | Louie et al. (2018) | Enhancing empathy: a role for virtual reality? | Non-medical student participants. |
