# Supplementary material for: A scoping review on the use of virtual patients for enhancing empathy in medical students
Source: Med Educ Online. 2025 Dec 25;31(1):2607825. doi: 10.1080/10872981.2025.2607825 (PMC12777885; doi:10.1080/10872981.2025.2607825)
Supplement: Additional file 2.docx [file ZMEO_A_2607825_SM7672.docx]

Additional file 2: Search strategy for databases

| **No.** | **Query** | **Results** |
| --- | --- | --- |
| #1 | “Students, Medical” [MeSH] OR “medical student*” [TW] OR “Education, Medical” [MeSH] OR “medical educat*” [TW] OR  “undergraduate medical student*” [TW] OR “undergraduate medical education*” [TW] OR “medical education curriculum*” [TW] OR “medical teach*” [TW] OR “medical train*” [TW] | 265,216 |
| #2 | “empathy” [MeSH] OR “empath*” [TW] | 40,759 |
| #3 | “Virtual reality” [MeSH] OR “virtual reality*” [TW] OR VR [TW] OR “virtual patient*” [TW] OR “virtual environment*” [TW] OR “virtual world*” [TW] OR “avatar*” [TW] OR “virtual human*” [TW] OR “VPs” [TW] | 39,611 |
| #4 | #1 AND #2 AND #3 | 49 |

Database #1: PubMed (MEDLINE)

Search date: January 31, 2025

Database #2: CINAHL

Search date: February 10, 2025

| **No.** | **Query** | **Results** |
| --- | --- | --- |
| S1 | MH “students, medical” OR AB “medical student*” OR MH “education, medical” OR AB “medical educat*” OR AB “undergraduate medical education” OR AB 2medical education curriculum*” OR AB “medical teach*” OR AB “medical train*” | 59,231 |
| S2 | MH “empathy” OR AB “empath*” | 20,679 |
| S3 | MH “virtual reality” OR AB “virtual real*” OR AB VR OR AB “virtual patient*” OR AB “virtual environment*” OR AB “virtual world*” OR AB “avatar*” OR AB “virtual human*” OR AB “VPs” | 12,553 |
| S4 | S1 AND S2 AND S3 | 22 |

Database #3: Education Resources Information Center: ERIC

Search date: February 10, 2025

| **No.** | **Query** | **Results** |
| --- | --- | --- |
| S1 | DE “Medical Students” OR KW “medical student*” OR DE “Medical Education” OR KW “medical educat*” OR KW  “undergraduate medical student*” OR KW “undergraduate medical education*” OR KW “medical education curriculum*” OR KW “medical teach*” OR KW “medical train*” | 13,095 |
| S2 | DE “empathy” OR KW “empath*” | 6,678 |
| S3 | DE “Computer Simulation” OR KW “computer simulation *” OR KW VR OR KW “virtual patient*” OR KW “virtual environment*” OR KW “virtual world*” OR KW “avatar*” OR KW “virtual human*” OR KW “VPs” | 10,792 |
| S4 | S1 AND S2 AND S3 | 1 |

Database #4: Web of Science

Search date: February 5, 2025

| **No.** | **Query** | **Results** |
| --- | --- | --- |
| #1 | TS= (“medical students” OR “medical student” OR “medical education” OR “medical educat*” OR “undergraduate medical student*” OR “undergraduate medical education*” OR “medical education curriculum*” OR “medical teach*” OR “medical train*”) | 111,922 |
| #2 | TS= (“empathy” OR “empath*”) | 50,401 |
| #3 | TS= (“virtual reality” OR “virtual reality*” OR VR OR “virtual patient*” OR “virtual environment*” OR “virtual world*” OR “avatar*” OR “virtual human*” OR “VPs”) | 86,651 |
| #4 | #1 AND #2 AND #3 | 78 |

Database #5: Scopus

Search date: February 10, 2025

| **No.** | **Query** | **Results** |
| --- | --- | --- |
| S1 | [TAK] “Students, Medical” OR [TAK] “medical student*” OR [TAK] “Education, Medical” OR [TAK] “medical educat*” OR [TAK] “undergraduate medical student*” OR [TAK] “undergraduate medical education*” OR [TAK] “medical education curriculum*” OR [TAK] “medical teach*” OR [TAK] “medical train*” | 409,535 |
| S2 | [TAK] “empathy” OR [TAK] “empath*” | 88,327 |
| S3 | [TAK] “Virtual reality” OR [TAK] “virtual reality*” OR [TAK] VR OR [TAK] “virtual patient*” OR [TAK] “virtual environment*” OR [TAK] “virtual world*” OR [TAK] “avatar*” OR [TAK] “virtual human*” OR [TAK] “VPs” | 252,661 |
| S4 | S1 AND S2 AND S3 | 84 |

Database #6: Cochrane Library

Search date: February 12, 2025

| **No.** | **Query** | **Results** |
| --- | --- | --- |
| #1 | [mh "Students, Medical"] OR (medical NEXT student*) OR [mh "Education, Medical"] OR (medical NEXT educat*) OR (undergraduate NEXT medical NEXT student*) OR (undergraduate NEXT medical NEXT education*) OR (medical NEXT education NEXT curriculum*) OR (medical NEXT teach*) OR (medical NEXT train*) | 13,612 |
| #2 | [mh empathy] OR (empath*) | 3,398 |
| #3 | [mh "Virtual reality"] OR (virtual NEXT reality*) OR (VR) OR (virtual NEXT patient*) OR (virtual NEXT environment*) OR (virtual NEXT world*) OR (avatar*) OR (virtual NEXT human*) OR (VPs) | 12,265 |
| #4 | #1 AND #2 AND #3 | 23 |
