# Supplementary material for: A scoping review on the use of virtual patients for enhancing empathy in medical students
Source: Med Educ Online. 2025 Dec 25;31(1):2607825. doi: 10.1080/10872981.2025.2607825 (PMC12777885; doi:10.1080/10872981.2025.2607825)
Supplement: Additional file 3.docx [file ZMEO_A_2607825_SM7673.docx]

Additional file 3. Data extraction form

| Categories | Type of data |
| --- | --- |
| 1. Study characteristics | First author |
|  | Year of publication |
|  | Country of study |
|  | Purpose |
|  | Research design |
|  | Population and sample size |
|  | Findings regarding medical students’ empathy |
| 2. Research questions | Definition of empathy (definition source and empathy components) * |
|  | Clinical scenarios (primary diseases, associated symptoms or comorbidities, and social contexts) * |
|  | Technology modalities of virtual patients (immersive, desktop-based, mobile-based) *  Educational design (duration, frequency, interval) * |
|  | Empathy enhancement evaluation (quantitative measures and their timing, and qualitative assessments and their timing) * |

* Fields that were added or modified compared to the preliminary extraction form of the protocol
